# Regulation of glycine metabolism by the glycine cleavage system and conjugation pathway in mouse models of non‐ketotic hyperglycinemia

**DOI:** 10.1002/jimd.12295

**Published:** 2020-08-11

**Authors:** Kit‐Yi Leung, Sandra C. P. De Castro, Chloe Santos, Dawn Savery, Helen Prunty, Diana Gold‐Diaz, Stuart Bennett, Simon Heales, Andrew J. Copp, Nicholas D. E. Greene

**Affiliations:** ^1^ Great Ormond Street Institute of Child Health, University College London London UK; ^2^ Department of Chemical Pathology Great Ormond Street Hospital for Children NHS Foundation Trust London UK

**Keywords:** benzoate, cinnamate, glycine cleavage system, glycine conjugation, glycine decarboxylase, non‐ketotic hyperglycinemia

## Abstract

Glycine abundance is modulated in a tissue‐specific manner by use in biosynthetic reactions, catabolism by the glycine cleavage system (GCS), and excretion via glycine conjugation. Dysregulation of glycine metabolism is associated with multiple disorders including epilepsy, developmental delay, and birth defects. Mutation of the GCS component glycine decarboxylase (GLDC) in non‐ketotic hyperglycinemia (NKH) causes accumulation of glycine in body fluids, but there is a gap in our knowledge regarding the effects on glycine metabolism in tissues. Here, we analysed mice carrying mutations in *Gldc* that result in severe or mild elevations of plasma glycine and model NKH. Liver of Gldc‐deficient mice accumulated glycine and numerous glycine derivatives, including multiple acylglycines, indicating increased flux through reactions mediated by enzymes including glycine‐*N*‐acyltransferase and arginine: glycine amidinotransferase. Levels of dysregulated metabolites increased with age and were normalised by liver‐specific rescue of *Gldc* expression. Brain tissue exhibited increased abundance of glycine, as well as derivatives including guanidinoacetate, which may itself be epileptogenic. Elevation of brain tissue glycine occurred even in the presence of only mildly elevated plasma glycine in mice carrying a missense allele of *Gldc*. Treatment with benzoate enhanced hepatic glycine conjugation thereby lowering plasma and tissue glycine. Moreover, administration of a glycine conjugation pathway intermediate, cinnamate, similarly achieved normalisation of liver glycine derivatives and circulating glycine. Although exogenous benzoate and cinnamate impact glycine levels via activity of glycine‐*N*‐acyltransferase, that is not expressed in brain, they are sufficient to lower levels of glycine and derivatives in brain tissue of treated Gldc‐deficient mice.

## 1 INTRODUCTION

Glycine, the smallest amino acid, functions as a neurotransmitter, a one‐carbon donor in folate one‐carbon metabolism and a precursor in biosynthesis of proteins and other molecules. The cell and tissue‐specific abundance of glycine depends on the balance between its use in biosynthetic reactions, catabolism by the glycine cleavage system (GCS) and excretion via the glycine conjugation pathway.

**FIGURE 1:**
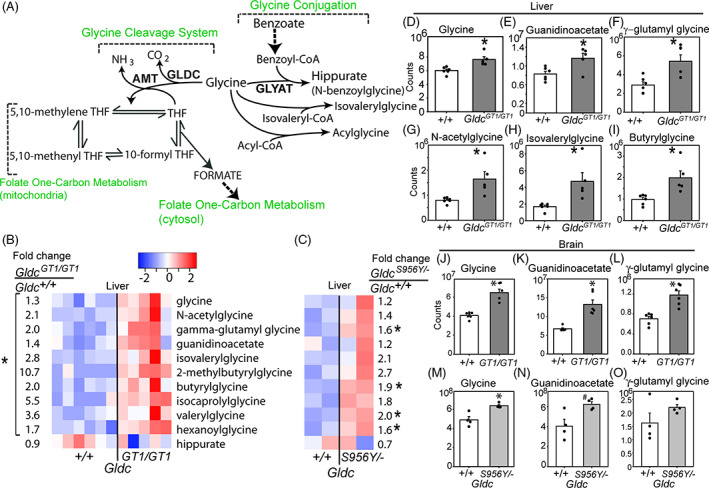
Loss of function of the glycine cleavage system leads to increased levels of glycine derivatives and glycine conjugation activity at post‐natal day 7 (P7). A, Diagram showing routes of hepatic glycine metabolism. B,C, Heatmaps showing glycine and glycine derivatives including acylglycines that differ significantly in scaled abundance in liver of *Gldc* ^*GT1/GT1*^ compared with *Gldc* ^*+/+*^ mice at P7. Each column represents an individual mouse, with colour intensity indicating lower (blue)/higher (red) abundance. Fold‐changes show relative abundance in B, *Gldc* ^*GT1/GT1*^ (n = 5) vs *Gldc* ^+/+^ (n = 6) and (C) *Gldc* ^*S956Y/−*^ (n = 2) vs *Gldc* ^+/+^ (n = 2) mice. D‐I, Abundance of glycine and selected derivatives in liver: mass spectrometry counts. J‐O, Abundance of J, glycine; K, guanidinoacetate; and L, γ‐glutamylglycine in brain of *Gldc* ^*GT1/GT1*^ mice (n = 6 per genotype). The brain of *Gldc* ^*S956Y/−*^ mice (n = 4 per genotype) also exhibits elevated abundance of M, glycine and N‐O, trends towards increases in guanidinoacetate and γ‐glutamylglycine (significantly different t corresponding wild‐type; *P < .05, #*P* < .1, Welch's *t*‐test)

The GCS comprises glycine decarboxylase (GLDC), amino methyltransferase (AMT), GCS H‐protein (GCSH), and dihydrolipoyl dehydrogenase (DLD).^1^ It is localised in the mitochondrial matrix where it acts to decarboxylate glycine, with release of CO_2_ and concomitant transfer of a one‐carbon group to tetrahydrofolate (Figure 1A). Mutation of *GLDC* or *AMT* causes non‐ketotic hyperglycinemia (NKH), a severe life‐limiting autosomal recessive neurometabolic disorder characterised by accumulation of excess glycine in body fluids and tissue, epilepsy and profound developmental delay.^2^, ^3^

In addition to ingestion, use in biosynthetic reactions and catabolism by the GCS, glycine abundance is modulated by use in conjugation reactions, primarily mediated by glycine‐*N*‐acyltransferase (*GLYAT*) in the liver and kidneys.^4^, ^5^ The glycine conjugation system has been proposed to function primarily as a mechanism for removal of benzoate, phenylpropionate, and other aromatic amino acids, produced from dietary polyphenols by gut microbes.^5^ These aromatic acids are detoxified by conversion to acyl‐CoA‐thioesters, which can be conjugated with glycine to form *N*‐acylglycines (Figure 1A) or undergo β‐oxidation to benzoyl‐CoA. GLYAT‐mediated conjugation of benzoyl‐CoA in the mitochondrial matrix irreversibly generates hippurate (*N*‐benzoylglycine) which is excreted (Figure 1A). In parallel with the concepts of glycine conjugation of aromatic acids an alternative, but not mutually exclusive, hypothesis proposes that the main function of glycine conjugation reactions is deportation of glycine as a means of regulating neurotransmitter levels.^4^ This is based on the premise that lowering the tissue pool of glycine in liver and kidneys would in turn lower glycine concentrations in plasma, CSF and brain. Adults are estimated to excrete around 400 to 800 mg per day of glycine via the conjugation pathway,^4^ activity of which is influenced both by diet and composition of the gut microbiota.^5^, ^6^, ^7^

The conversion of ingested benzoic acid (a precursor of benzoyl‐CoA) to urinary hippuric acid is thought to be the first‐described metabolic reaction in humans.^8^, ^9^, ^10^, ^11^ Similarly, it was recognised that excretion of hippurate was increased following ingestion of glycine.^10^ The tractability of glycine conjugation, leading to increased excretion of hippurate following either glycine or benzoate ingestion, has been exploited in treatment of NKH since the disease was recognised in the 1960s.^11^, ^12^ Administration of sodium benzoate lowers glycine in blood and CSF of NKH patients and can help control epilepsy, but does not ameliorate developmental delay.^13^, ^14^

While plasma and CSF provide an indication of glycine abundance, there is a gap in our knowledge regarding the consequences of GCS loss of function for glycine metabolism within tissues. Similarly, the extent to which tissue metabolite concentrations are normalised in NKH by addition of exogenous substrates for glycine conjugation, such as benzoate, is not known. In order to address these questions, we analysed mice in which GCS activity is suppressed by *Gldc* mutation, providing models for NKH.

## 2 RESULTS

### 2.1 Loss of GCS activity causes accumulation of glycine and glycine conjugates in the liver

Loss of function of GLDC in individuals with NKH and in mice causes increased glycine concentration in body fluids, but the corresponding effects on tissue content of glycine and related metabolites have not been determined. In particular, the extent to which glycine conjugation may be activated by presence of excess tissue glycine is unknown. Mice that are homozygous for the *Gldc* ^*GT1*^ gene‐trap allele exhibit approximately 90% reduction of *Gldc* mRNA expression, loss of GCS activity, elevated glycine in plasma and urine and premature lethality.^15^ Glycine is already elevated pre‐natally in *Gldc* ^*GT1/GT1*^ embryos^15^ while NKH presents in neonates or young infants, similarly suggesting an early accumulation of glycine in humans with impaired GCS activity.

In order to compare glycine handling in *Gldc*‐deficient (*Gldc* ^*GT1/GT1*^) and wild‐type (*Gldc* ^+/+^) post‐natal tissues, we used a platform comprising four mass spectrometry methods that together allow relative quantification of glycine and a series of glycine conjugates. Liver was initially analysed, being the major site of glycine cleavage and glycine conjugation enzymatic activity. At post‐natal day 7 (P7), the liver of *Gldc*‐deficient (*Gldc* ^*GT1/GT1*^) mouse pups showed significantly elevated levels of glycine (Figure 1B,D) and several glycine derivatives. These included guanidinoacetate and γ‐glutamyl glycine (Figure 1B,E,F), which are formed by combination of glycine with glutamate and arginine by l‐Arginine:glycine amidinotransferase (AGAT) and gamma‐glutamyl transpeptidase (GGT), respectively. A number of *N*‐acylglycine molecules were also significantly increased in abundance (Figure 1B) including valerylglycine, hexanoylglycine, isovalerylglycine, and butyrylglycine (Figure 1H‐I), reflecting the broad specificity of GLYAT towards acyl‐CoA esters.^16^ We conclude that the glycine conjugation system is active in young, pre‐weaning, mice and exhibits increased activity in the presence of excess glycine.

### 2.2 Accumulation of glycine in the brain of *Gldc‐*deficient mice

We next asked whether the metabolites that accumulate in the liver of *Gldc* ^*GT1/GT1*^ mice are also present in brain tissue, an important site of pathophysiology in NKH patients. Glycine, guanidinoacetate and γ‐glutamyl glycine were each significantly more abundant in brain of *Gldc*‐deficient mice than in wild‐type mice at P7 (Figure 1J‐L). The genes encoding AGAT and GGT are both expressed in brain, suggesting that presence of elevated guanidinoacetate and γ‐glutamylglycine concentrations could result from production within the brain. In contrast, acylglycine conjugates were not detected in brain.

### 2.3 Partial loss of GCS activity

In addition to analysis of *Gldc* ^*GT1/GT1*^ mice, in which liver GCS activity is below the limit of detection,^15^ we investigated the effect of partial loss of Gldc function in order to determine whether glycine derivatives still accumulate in tissues. We generated a novel mouse line carrying the *Gldc* ^*S956Y*^ allele. The mouse p.S956Y mutation is equivalent to the human p.S951Y mutation, previously found in a patient with NKH who was a compound heterozygote for deletion of exon 1.^3^ To recreate this situation we generated compound heterozygous *Gldc* ^*S956Y/−*^ mice (by intercross of *Gldc* ^*S956Y/*+^ and *Gldc* ^+/−^ mice). Adult *Gldc* ^*S956Y/−*^ mice showed mild but significant elevation of plasma glycine compared with wild‐type littermates (445 vs 370 μM; *P* < .05, *t*‐test), whereas heterozygosity for either allele did not result in elevated plasma glycine (Supplementary Figure S1A). This elevation in glycine was not associated with premature lethality; the *Gldc* ^*S956Y/−*^ genotype accounted for 27% of mice at 5 to 6 weeks (n = 17/62 mice among 9 litters), which did not differ from the expected Mendelian ratio (25%).

The relatively mild elevation of plasma glycine in *Gldc* ^*S956Y/−*^ mice compared with the major increase in *Gldc* ^*GT1/GT1*^ mice (900‐1000 μM),^15^ indicates that p.S956Y retains some enzymatic activity. Nevertheless, analysis of *Gldc* ^*S956Y/−*^ liver samples at P7 revealed accumulation of γ‐glutamyl glycine and acylglycines as found in *Gldc* ^*GT1/GT1*^ mice, albeit to a lesser extent (Figure 1C). Notably, despite the modestly elevated plasma glycine, the brain of P7 *Gldc* ^*S956Y/−*^ mice exhibited significant accumulation of glycine (1.3‐fold) and trends towards accumulation of guanidinoacetate (1.5‐fold) and γ‐glutamyl glycine (1.4‐fold; Figure 1M‐O; Figure Supplementary Figure S1B).

We conclude that excess glycine leads to increased flux through reactions mediated by several enzymes in liver that use glycine as substrate including GLYAT, AGAT, and GGT. However, this is insufficient to maintain physiological glycine concentration in liver tissue. Moreover, hepatic glycine conjugation does not prevent elevation of plasma glycine when GCS activity is diminished, nor prevent accumulation of glycine and its derivatives in brain.

### 2.4 Accumulation of glycine derivatives progresses with age and is normalised by restoration of hepatic glycine cleavage activity

**FIGURE 2:**
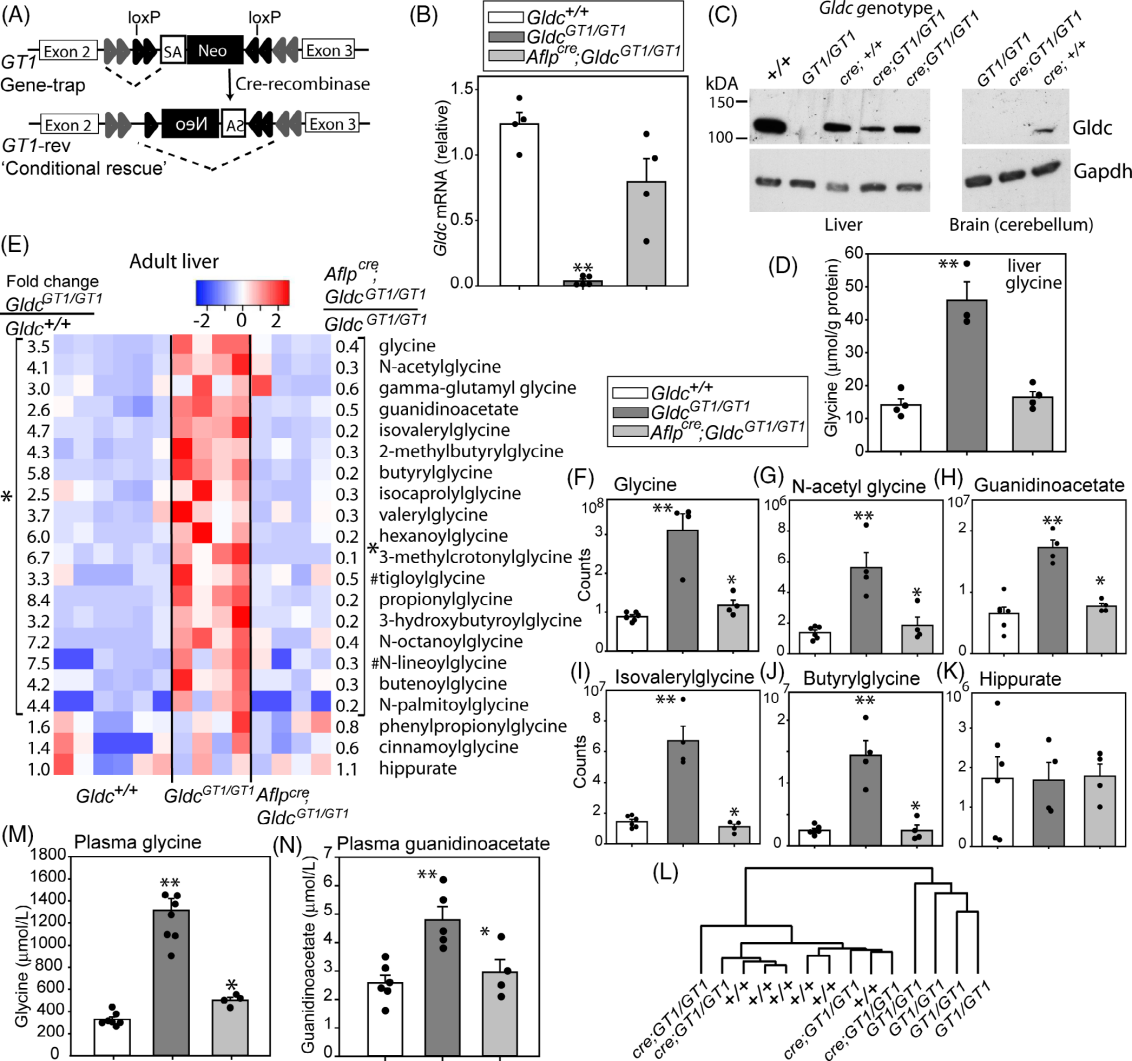
Regulation of glycine and glycine derivatives by the hepatic glycine cleavage system. A, Gene‐trapping of *Gldc* at the *Gldc* ^*GT1*^ allele is reversible by cre‐mediated recombination of the inverted loxP sites, generating a ‘conditional rescue’ allele in which the splice acceptor (SA) of the trap is in an inactive orientation. B, mRNA expression is restored in liver of *Gldc* ^*GT1/GT1*^ mice following hepatocyte‐specific expression of cre recombinase (under control of the albumin and alpha‐fetoprotein regulatory elements). C, Immunoblot confirms that Gldc protein expression is restored in liver but not in brain of *Aflp* ^*cre*^;*Gldc* ^*GT1/GT1*^ mice, as compared with *Gldc* ^*GT1/GT1*^ mice in which Gldc is below the limit of detection (Gapdh was used as a loading control). D, Glycine abundance is increased in liver of adult *Gldc* ^*GT1/GT1*^ mice and normalised by reinstatement of hepatic *Gldc* expression (**significant difference from *Gldc* ^+/+^; *P* < .01, ANOVA). E, Heatmap shows relative abundance of glycine and glycine derivatives in liver of adult wild‐type (n = 6), Gldc‐deficient (n = 4) and hepatic Gldc‐rescued (n = 4) mice. Each column represents an individual mouse. Fold‐changes show relative abundance in *Gldc* ^*GT1/GT1*^ vs *Gldc* ^+/+^ (left side of panel) and *Aflp* ^*cre*^;*Gldc* ^*GT1/GT1*^ vs *Gldc* ^*GT1/GT1*^ (right side of panel). All fold‐changes within brackets show significant difference between genotypes (*P < .05; paired *t* test) except where indicated (#*P* < .1). F‐K, Abundance of glycine and selected glycine derivatives (arbitrary scale, representing raw counts from mass spectrometry); ** different from *Gldc* ^+/+^, *different to constitutive *Gldc* ^*GT1/GT1*^; *P* < .05, ANOVA). K, Clustering of samples based on relative abundance of the glycine metabolites in panel E groups hepatic‐rescued mice with wild‐types and distinct from constitutive *Gldc* ^*GT1/GT1*^ mice. L,M, Reinstatement of *Gldc* expression in liver is associated with normalisation of plasma glycine and guanidinoacetate concentrations in *Aflp* ^*cre*^;*Gldc* ^*GT1/GT1*^ mice

We asked whether glycine conjugates progressively accumulate by analysing adult mice, at 6 weeks of age. In parallel, we asked whether tissue‐specific reinstatement of Gldc function in the liver is sufficient to normalise altered hepatic metabolism and/or circulating glycine in *Gldc‐*deficient mice. The gene‐trap *Gldc* ^*GT1*^ construct is flanked by inverted loxP sites that can be recombined by cre recombinase leading to reversal of the orientation (Figure 2A), such that the splice acceptor is no longer in the ‘trap’ configuration (confirmed by genomic PCR). Conditional rescue of *Gldc* expression in the liver was thereby achieved by introduction of a hepatocyte‐specific cre (*AlfpCre*) allele and confirmed by qRT‐PCR (Figure 2B) and immunoblot (Figure 2C). Functional restoration of hepatic GCS activity in *AlfpCre*; *Gldc* ^*GT1/GT1*^ mice was confirmed by analysis of glycine, which was present at increased abundance in liver tissue of adult *Gldc* ^*GT1/GT1*^ mice and corrected by liver‐specific rescue of *Gldc* expression (Figure 2D).

In addition to glycine, the liver of adult *Gldc‐*deficient mice (6 weeks of age) exhibited significant accumulation of glycine derivatives as found at P7 (Figure 2E‐J). In addition to the six acylglycines that accumulated in young mice, a further 10 acylglycines also exhibited significantly increased abundance in liver of adult *Gldc‐*deficient mice (Figure 2E). The magnitude of elevation of glycine and its derivatives in *Gldc*‐deficient adults compared with wild‐type was greater than in P7 pups, suggesting accumulation from pre‐weaning to adult stages. Moreover, many glycine derivatives that showed significant changes in Gldc‐deficient liver were elevated to a greater extent than glycine itself, suggesting that production of these compounds is not rate‐limiting and/or that these metabolites reflect active mechanisms to remove excess glycine. Interestingly, the abundance of hippurate was not elevated in *Gldc* ^*GT1/GT1*^ liver, suggesting limited availability of benzoyl‐CoA (Figure 2K).

Abundance of each of the glycine‐derivatives that accumulated in *Gldc‐*deficient liver was corrected in *AlfpCre*; *Gldc* ^*GT1/GT1*^ mice (Figure 2E‐J). Analysis based on these 20 metabolites clustered the ‘tissue‐specific rescue’ samples with wild‐type liver, and distinct from liver of constitutive *Gldc‐*deficient mice (Figure 2L). These findings confirm that hepatic GCS activity regulates abundance of liver glycine derivatives in a tissue autonomous manner.

Expression of *Gldc* solely in the liver in *AlfpCre*; *Gldc* ^*GT1/GT1*^ mice was sufficient to maintain plasma glycine at a similar concentration as in wild‐type mice (Figure 2M). In addition to glycine, plasma of adult Gldc‐deficient mice also showed elevated concentration of the glycine/arginine derivative guanidinoacetate (Figure 2N), as in tissue, and this was normalised by liver‐specific rescue of *Gldc* expression. Hence, hepatic GCS activity can maintain circulating glycine and guanidinoacetate levels, even when *Gldc* expression is absent in other tissues.

### 2.5 Exogenous benzoate modulates abundance of circulating and hepatic glycine derivatives

In *Gldc‐*deficient mice, endogenous glycine conjugation activity is insufficient to prevent elevation of plasma glycine levels. We therefore tested the effect of exogenous benzoate, a precursor of benzoyl‐CoA which is a substrate for GLYAT‐mediated glycine conjugation in liver (Figure 1A). In a preliminary study in *Gldc* ^*GT1/GT1*^ mice we found that administration of sodium benzoate in drinking water (35 mM for 7 days) was sufficient to lower plasma glycine concentration (1170 ± 311 vs 398 ± 35 μM, n = 4 *Gldc* ^*GT1/GT1*^ per treatment group) to a level comparable to that in *AlfpCre*; *Gldc* ^*GT1/GT1*^ mice. Plasma guanidinoacetate was also lowered by benzoate treatment (Supplementary Figure S2).

In Gldc‐deficient mice and NKH patients, the lowering of plasma glycine with benzoate treatment implies that provision of additional substrate, leads to increased glycine conjugation activity by GLYAT. However, the effect on levels of glycine and derivatives in tissue has not been determined. Nor is it known whether conjugation of glycine with benzoyl‐CoA occurs in preference to conjugation of glycine with other acyl‐CoA molecules or whether abnormal accumulation of acylglycines in *Gldc*‐deficient liver can be reversed by benzoate treatment. Hence, we tested whether benzoate‐mediated activation of glycine conjugation was sufficient to normalise abundance of glycine derivatives within liver tissue.

**FIGURE 3:**
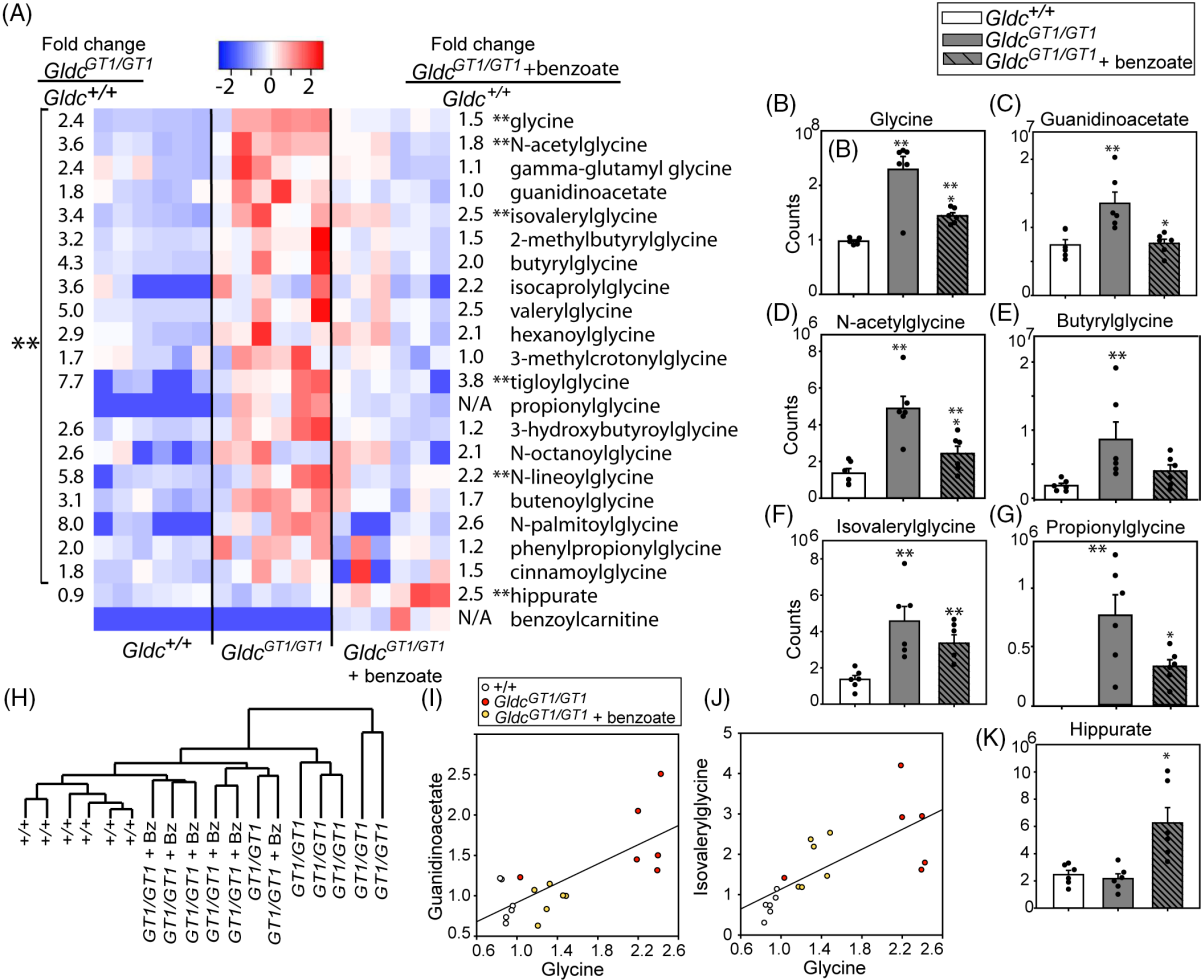
Stimulation of glycine conjugation regulates hepatic glycine metabolism. A‐G, Accumulation of glycine and derivatives in liver of *Gldc‐*deficient mice is partially corrected by benzoate administration. A, Heatmap and fold‐changes for glycine and glycine derivatives in liver of adult wild‐type and *Gldc* ^*GT1/GT1*^ mice with/without administration of benzoate (n = 6 per group). B‐G, Mass spectrometry data for glycine and selected derivatives. H, Clustering on the basis of abundance of glycine derivatives in liver separates benzoate‐treated *Gldc* ^*GT1/GT1*^ from untreated *Gldc* ^*GT1/GT1*^. I‐J, Relationship between glycine abundance (scaled) and I, guanidinoacetate or J, isovalerylglycine. K, Benzoate treatment leads to significant accumulation of hippurate in liver (**significantly different from wild‐type, *P* < .05; *significantly different from untreated *Gldc* ^*GT1/GT1*^, *P* < .05 ANOVA)

We confirmed up‐regulation of the panel of glycine derivatives that accumulated in Gldc‐deficient adult liver in a second cohort of +/+ and *Gldc* ^*GT1/GT1*^ mice (n = 5 per genotype; Figure 3A‐G). In a parallel group of benzoate‐treated *Gldc* ^*GT1/GT1*^ mice, we found significant lowering of the abundance of glycine, guanidinoacetate and *N*‐acetylglycine in liver (Figure 3A‐D). The abundance of several acylglycine molecules was also significantly lowered (2‐methylbutyrylglycine, *N*‐lineoylglycine) or trended lower (butyrylglycine, propionylglycine; Figure 3A,E,F). Overall, clustering on the basis of glycine and glycine derivatives grouped benzoate‐treated liver of *Gldc* ^*GT1/GT1*^ mice more closely with wild‐type than untreated mutants (Figure 3H). We observed some differential sensitivity to benzoate treatment. For example, guanidinoacetate is lowered to wildtype levels in benzoate‐treated liver, even though glycine remains slightly elevated (Figure 3I), whereas levels of acylglycines, such as isovalerylglycine appear to correlate more closely with glycine (Figure 3J).

We conclude that accumulation of acylglycines in liver is reversible by diversion of GLYAT towards using benzoyl‐CoA as substrate. This is consistent with the observed increase in abundance of hippurate (benzoylglycine; Figure 3K). We also noted accumulation of benzoylcarnitine in liver of benzoate‐treated mice (Figure 3A), which was below the limit of detection in wildtype and *Gldc* ^*GT1/GT1*^ mice. Benzoylcarnitine has previously been reported in urine of patients being treated with benzoate for hyperammonemia and NKH.^11^, ^17^, ^18^

### 2.6 Activation of the glycine conjugation pathway by additional precursors of benzoyl‐CoA

**FIGURE 4:**
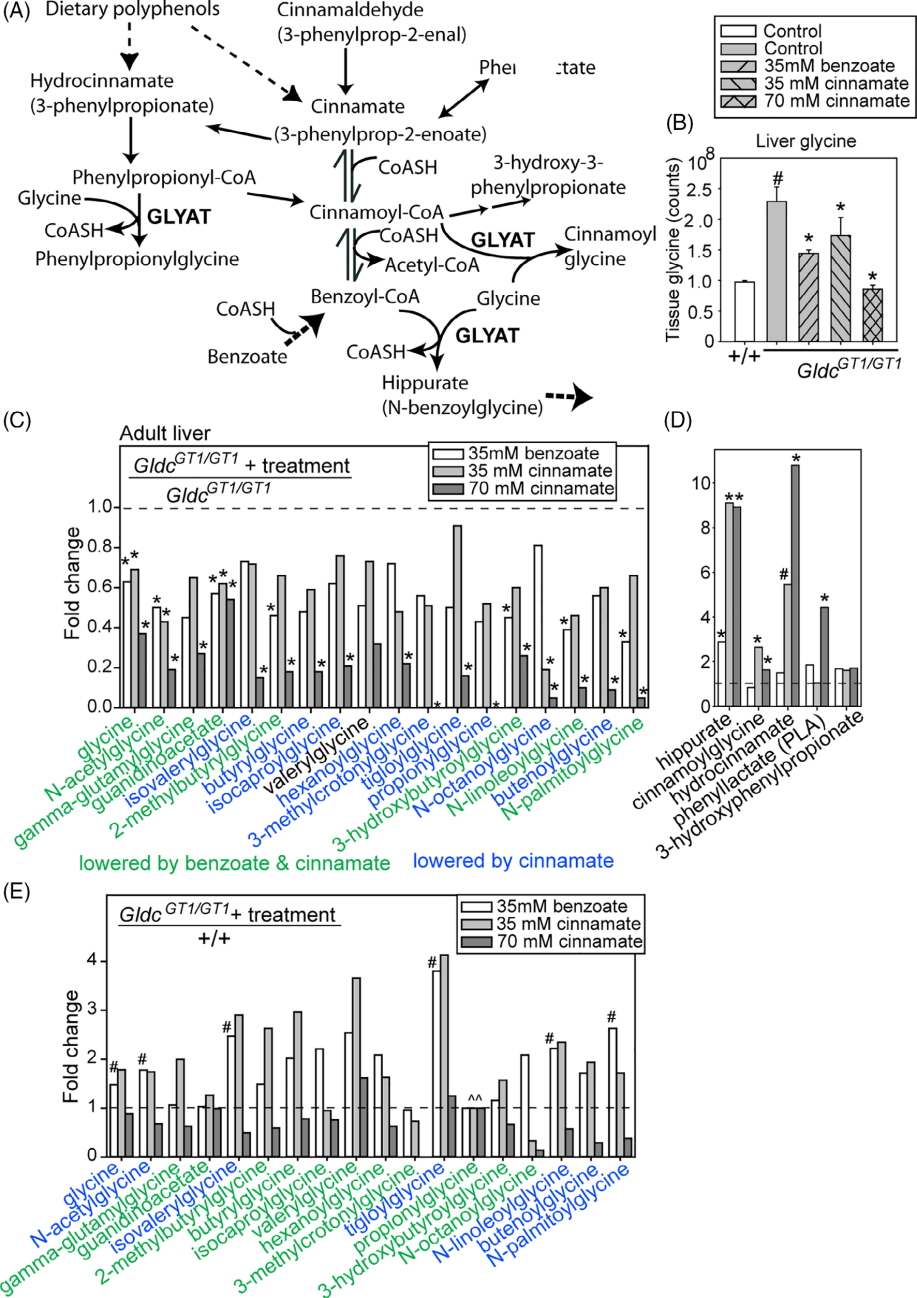
Benzoate and cinnamate stimulate increased hepatic glycine conjugation. A, The glycine conjugation pathway and relevant interconnected reactions. B, Abundance of glycine in liver of adult mice is significantly lowered by benzoate or cinnamate administration (# significantly different from *Gldc* ^+/+^, * significantly different from untreated *Gldc* ^*GT1/GT1*^, ANOVA). C‐E, Fold‐change of glycine metabolites in liver of benzoate‐ or cinnamate‐treated adult *Gldc* ^*GT1/GT1*^ mice compared with, C,D, untreated adult *Gldc* ^*GT1/GT1*^ or E, untreated wild‐type mice (n = 4‐6 per group). C, Glycine and its derivatives are lowered in abundance (below dashed line) by benzoate or cinnamate. D, Cinnamate or benzoate treatment is associated with increased abundance of specific glycine conjugates in adult liver (fold‐change above dashed line). E, Some derivatives are lowered to wild‐type levels indicated by dashed line, whereas glycine remains elevated in benzoate‐treated mice. (^̂) Propionylglycine was not detected in wild‐type or treated *Gldc* ^*GT1/GT1*^ mice. (^#^ significantly different from wild‐type, * significantly different from untreated *Gldc* ^*GT1/GT1*^, *P* < .05; ANOVA, n = 4‐5 per group)

Benzoyl‐CoA can be generated from exogenous benzoate or via endogenous β‐oxidation in the glycine conjugation pathway (Figure 4A). This pathway is proposed to represent a mechanism for removal of aromatic acids, produced from dietary polyphenols by gut microbes.^5^ Evidence of the potential for stimulation of this pathway by components other than exogenous benzoate came from the observation, as early as the 1840s, that hippurate is detected in urine following ingestion of cinnamic acid (3‐phenyl‐2‐propenoic acid).^19^, ^20^ We therefore hypothesised that cinnamate may act to lower glycine in *Gldc*‐deficient mice, as observed for benzoate. Consistent with this concept, we observed significantly lower abundance of glycine in liver of *Gldc* ^*GT1/GT1*^ mice treated with sodium cinnamate than in untreated controls (Figure 4B). The dose of 35 mM cinnamate is equimolar to that tested for benzoate and an additional cohort were administered 70 mM cinnamate.

We tested whether cinnamate treatment was also sufficient to normalise abundance of glycine‐containing molecules in *Gldc*‐deficient mice. Like glycine, the abundance of *N*‐acetylglycine, γ‐glutamylglycine, and guanidinoacetate were lowered by benzoate or cinnamate treatment in liver of *Gldc* ^*GT1/GT1*^ mice (Figure 4C; fold‐change below 1.0 compared with untreated *Gldc* ^*GT1/GT1*^ mice). The normalisation of metabolite abundance was also assessed by comparison to untreated wild‐type liver (Figure 4E), which revealed that each treatment lowered guanidinoacetate abundance to wild‐type levels, whereas glycine remained at significantly greater abundance than in wild‐type liver in benzoate‐treated *Gldc* mutants (Figure 4E). Similarly, the acylglycines that were more abundant in *Gldc* ^*GT1/GT1*^ than wild‐type liver showed a lower trend after benzoate or cinnamate treatment at 35 mM, and most were significantly lowered at the 70 mM dose of cinnamate (Figure 4C,E). Hence, cinnamate exhibits a dose‐response effect (Figure 4B,C), while equimolar benzoate and cinnamate have comparable overall effects on acylglycine abundance. In parallel with lowering of glycine and glycine derivatives, treatment with cinnamate increased the abundance of cinnamoylglycine, hydrocinnamate (3‐phenylpropionate), and phenyllactate (Figure 4D).

**FIGURE 5:**
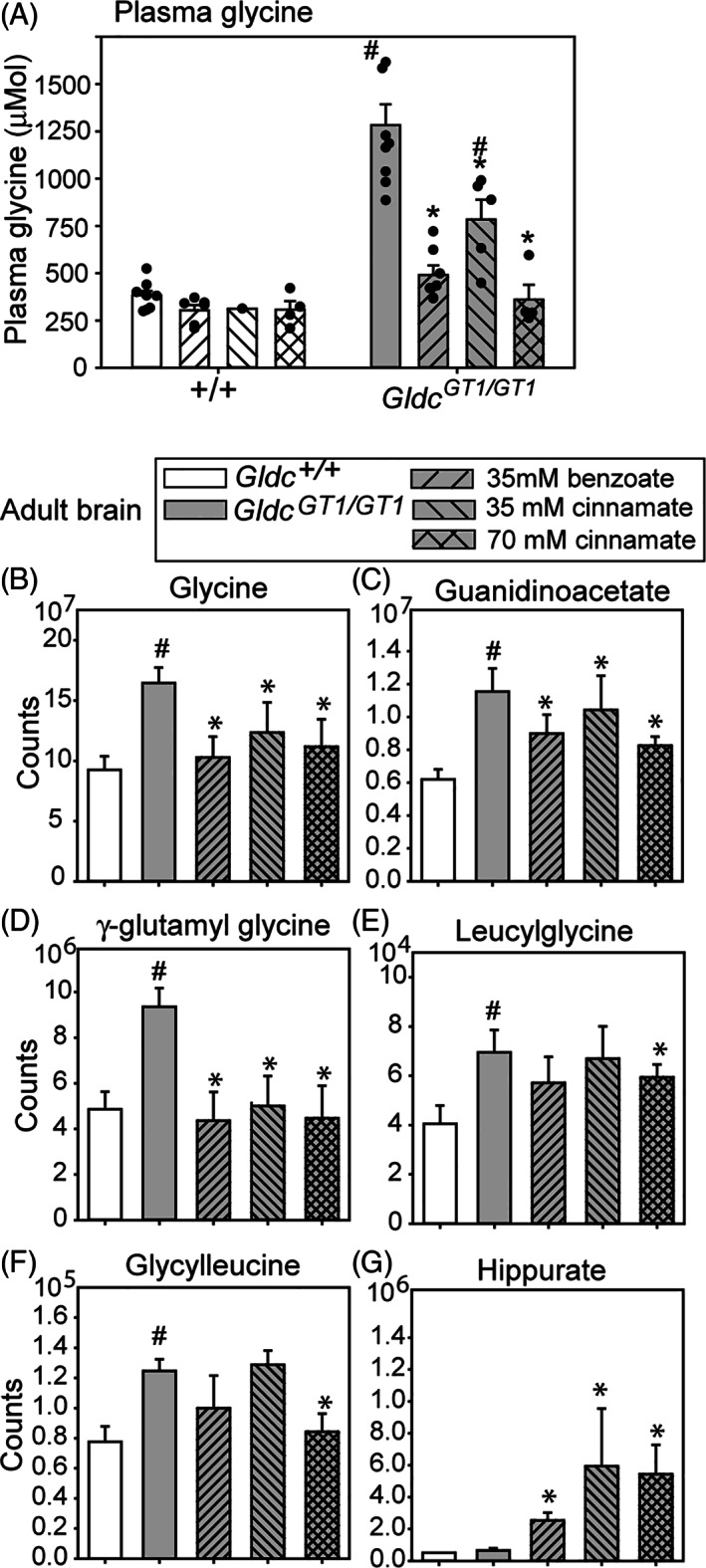
Regulation of circulating and brain tissue glycine by benzoate and cinnamate. A, Oral administration of benzoate or cinnamate significantly lowers plasma glycine concentration in adult *Gldc* ^*GT1/GT1*^ wild‐mice but not in wild‐type mice. B‐F, Glycine and glycine derivatives accumulate in brain of adult *Gldc* ^*GT1/GT1*^ mice, with normalisation following benzoate or cinnamate treatment. G, In contrast, while hippurate is detectable in brain of untreated mice, its abundance is significantly increased following benzoate or cinnamate treatment (^#^significantly different from wild‐type, * significantly different from untreated *Gldc* ^*GT1/GT1*^, *P* < .05; ANOVA, n = 4‐5 per group)

We asked whether stimulation of hepatic glycine conjugation lowered plasma glycine concentration. Benzoate and cinnamate administration did not significantly affect circulating glycine in wild‐type mice, but lowered glycine concentration in *Gldc*‐deficient mice (Figure 5A), with a dose‐dependent effect of cinnamate.

### 2.7 Accumulation of glycine and its derivatives in the brain of *Gldc‐*deficient mice and response to benzoate or cinnamate

As at P7 (Figure 1), we found that glycine, guanidinoacetate and γ‐glutamylglycine were each significantly more abundant in brain of *Gldc*‐deficient adult mice than in wild‐type mice (Figure 5B‐D). Accumulation of guanidinoacetate in *Gldc‐*deficient mice was accompanied by a mild elevation in creatinine in brain (and both creatine and creatinine in liver; Supplementary Figure S3).

Unlike at P7, *Gldc* ^*GT1/GT1*^ brain exhibited accumulation of glycine dipeptides by 6 weeks of age, including glycylleucine and leucylglycine (Figure 5E,F). Interestingly, these dipeptides were not elevated in *Gldc‐*deficient liver. Acylglycine conjugates (which are altered in liver) were not detected in the brain, consistent with reported lack of *GLYAT* expression in brain and suggesting the absence of another glycine acyltransferase activity. Moreover, acylglycines are not transported to the brain from peripheral tissues in appreciable quantities.

Treatment of *Gldc* ^*GT1/GT1*^ mice with benzoate and cinnamate led to significant reduction in the abundance of glycine in brain tissue (Figure 5B). Given the lack of glycine acyltransferase activity in brain this effect is presumed to result from depletion of circulating glycine. Notably, normalisation of brain tissue glycine was also associated with lower abundance of guanidinoacetate and γ‐glutamylglycine (Figure 5C,D), while the higher dose of cinnamate also lowered glycine dipeptides (Figure 5E,F). Interestingly, although not generated in the brain, hippurate was present at increased concentration following benzoate or cinnamate administration (Figure 5G). Similarly, unlike in brain of untreated wild‐type mice (n = 5), cinnamoylglycine was occasionally detected at low abundance in the brain of some *Gldc* ^*GT1/GT1*^ mice (1 of 5 untreated; 2 of 5 benzoate‐treated, and 4 of 8 cinnamate‐treated). These observations suggest that a low level of hippurate (benzoylglycine) and cinnamoylglycine can enter the brain tissue when present at increased levels systemically.

## 3 DISCUSSION

Analysis of the abundance of glycine metabolites reveals tissue‐specific and age‐dependent complexity in the response to excess glycine caused by loss of function of the GCS. Consistent with our previous finding of elevated tissue glycine in embryos lacking GCS activity, glycine was significantly more abundant in liver and brain of *Gldc*‐deficient mice at early post‐natal and adult stages, than in wild‐type mice. Excess glycine leads to increased activity of multiple enzymes that use glycine as substrate including AGAT (Supplementary Figure S3A) and GGT, as evidenced by accumulation of guanidinoacetate and γ‐glutamylglycine in liver and brain. The striking accumulation of acylglycines in liver of *Gldc* ^*GT1/GT1*^ mice suggests that GLYAT mediates conjugation of glycine with a range of acyl‐CoA molecules.

Activation of GLYAT was insufficient to maintain physiological glycine concentration in liver tissue or plasma of *Gldc‐*deficient mice and glycine derivatives accumulated in both liver and brain tissue even when plasma glycine was only mildly elevated. These findings suggest that the function of the glycine conjugation pathway is more likely required for regulation of aromatic acids than regulation of glycine abundance. In humans, the excretion of acylglycine conjugates in patients with organic acidemias confirms this role of glycine conjugation. Mutations of isovaleryl dehydrogenase or propionyl‐CoA carboxylase lead to accumulation of toxic isovaleryl‐CoA or propionyl‐CoA, with glycine conjugation leading to excretion of isovalerylglycine and propionylglycine, respectively. This is analogous to our finding of isovalerylglycine accumulation in Gldc‐deficient mice produced by conjugation of excess glycine with isovaleryl‐CoA.

The concentration range of glycine in plasma of *Gldc‐*deficient, *Gldc* ^*GT1/GT1*^, mice resembles that in severe NKH, while concentrations in *Gldc* ^*S956Y/−*^ mice, which genocopies an NKH patient, are in the range observed in attenuated NKH (this study^15^, ^21^). Hence, our observations in mice carrying loss of function alleles of *Gldc* may provide insight into the metabolic dysregulation in NKH.

Clinical features of NKH include neonatal apnea, epilepsy, and profound developmental delay. Excess glycine is thought to contribute to epilepsy phenotypes that arise in NKH, in particular owing to its action as a co‐agonist with glutamate at *N*‐methyl‐d‐aspartate (NMDA) receptors.^13^, ^22^ A decrease in seizure frequency and severity can be achieved by benzoate treatment to lower glycine, while use of NMDA receptor antagonists may have beneficial effects.^23^, ^24^

The identification of glycine derivatives accumulating in the brain of *Gldc*‐deficient mice suggest that additional metabolites, alongside glycine itself, may contribute to NKH pathogenesis. For example, γ‐glutamylglycine has a similar structure to GABA (gamma‐aminobutyric acid) and can act as an antagonist of excitatory amino acids, including glutamate.^25^, ^26^ Similar to our finding in *Gldc*‐deficient mice, guanidinoacetate accumulates in guanidinoacetate methyltransferase (GAMT) deficiency, which has overlapping features with NKH, including hypotonia and seizures.^27^ Deleterious effects of GAMT deficiency result from the consequent creatine (methylguanidinoacetate) depletion. However, the associated accumulation of guanidinoacetate is also thought to have neurotoxic effects, and contribute to severity and frequency of seizures.^28^, ^29^

In *Gldc‐*deficient mice, accumulation of guanidinoacetate is not accompanied by creatine depletion, as it results from increased synthesis, as opposed to diminished removal. Nevertheless, we hypothesise that the epileptogenic potential of guanidinoacetate^28^ could play a role in NKH, perhaps exacerbating effects of excess glycine. While comparison of the magnitude of guanidinoacetate accumulation in humans and mice should be treated with caution, we note that the plasma concentration in *Gldc*‐deficient mice was approximately twofold higher than in wild‐types. By comparison a cohort of GAMT deficiency patients exhibited elevation of plasma of guanidinoacetate ranging from two‐ to nineteen‐fold (average ninefold increase).^30^ It would be of potential interest to test whether arginine restriction and/or ornithine supplementation could contribute to lowering of guanidinoacetate in the mouse model, as used in GAMT deficiency. Our finding that the glycine lowering effects of benzoate and cinnamate can also lower guanidinoacetate, imply that increased flux through AGAT in *Gldc‐*deficient liver is compensated by greater flux through GLYAT (generating hippurate) together with the action of GAMT (generating creatine).

Our findings provide insight into the potential effects of sodium benzoate within tissues of individuals with NKH, for whom benzoate is used as a glycine lowering agent in clinical management.^13^, ^14^ While comparison of dosage between mouse and humans can be imprecise, we note that the benzoate dosage used in this study equates to approximately 1000 mg/kg/day which is comparable to the 500 to 750 mg/kg/day often used for treatment of NKH.^14^, ^23^ In *Gldc‐*deficient mice, administration of benzoate proved an effective way to increase glycine conjugation and thereby lower abundance of glycine (and derivatives) in liver, and consequently plasma and brain, as a secondary effect. This also implies a potential for beneficial effect of liver‐directed gene replacement therapy. For example, control of glycine could lower or remove the requirement for benzoate treatment, for control of epilepsy, and thereby avoid associated side‐effects.

We showed that glycine conjugation is also activated by cinnamate, as implied by the prescient work of Ure and Garrod which demonstrated the presence of hippurate in urine after cinnamic acid ingestion.^19^ Accumulation of cinnamoylglycine in liver following cinnamate treatment presumably results from GLYAT‐mediated use of cinnamoyl‐CoA as substrate. This reaction may be favoured in mice over the production of 3‐hydroxy‐3‐phenylpropionyl CoA which is reported to be favoured in rat.^31^ The increased abundance of hydroxycinnamate (3‐phenylpropionate) in cinnamate‐treated mice likely results from beta‐oxidation of cinnamate, which occurs in rat liver mitochondria but may also be mediated by gut microbes.^32^ Phenyllactate has been detected in liver in experimentally‐induced phenylketonuria, via production of phenylpyruvic acid, and may cross the blood‐brain barrier.^33^

Here, we found that the action of cinnamate in stimulating glycine conjugation was sufficient to normalise glycine, and many glycine derivatives, within the liver and brain of *Gldc*‐deficient mice. These findings are consistent with a mechanism in which cinnamate administration sequentially leads to intracellular production of cinnamoyl‐CoA, benzoyl‐CoA, and hippurate. Hence, there is scope for further longer‐term treatment studies that may lead to clinical testing of cinnamate to control the excess glycine abundance in diseases such as NKH.

## 4 MATERIALS AND METHODS

### 4.1 Mice

Animal studies were carried out under regulations of the Animals (Scientific Procedures) Act 1986 of the UK Government, and in accordance with the guidance issued by the Medical Research Council, UK in *Responsibility in the Use of Animals for Medical Research* (July 1993). *Gldc*‐deficient mice, denoted *Gldc* ^*GT1*^, carry a gene‐trap construct in intron 2 of *Gldc* ^15^. *Gldc* null (*Gldc* ^*GT2*^, denoted *Gldc* ^*−*^) carry a gene‐trap construct in intron 19.^34^ Hepatocyte‐specific targeting of the liver used transgenic mice expressing Cre recombinase under the control of mouse albumin and α‐fetoprotein regulatory elements (*AlfpCre*).^35^ The Gldc^S956Y^ mouse line (C57BL/6NTac‐Gldc^em1H^/H) was generated at MRC Harwell (A410) by CRISPR/Cas9 targeting. The S956Y variant was encoded by nucleotide substitution c.3025C>A (see Supporting Information for additional details).

### 4.2 Sample collection

Blood was collected by terminal cardiac exsanguination under isofluorane anaesthetic, transferred to lithium‐heparin tubes (BD Microtainer), immediately centrifuged for isolation of plasma and stored at −20°C). Tissues were collected immediately after sacrifice, rinsed in phosphate buffered saline (PBS), snap frozen on dry ice and stored at −80°C (for RNA and metabolite analysis).

### 4.3 qRT‐PCR

The abundance of *Gldc* mRNA in mice carrying the *Gldc* ^*GT1*^ allele was analysed by qRT‐PCR, using primers located in exons 2 and 4 that amplify the wild‐type but not the mutant transcript.

### 4.4 Mouse treatments

Sodium benzoate and sodium cinnamate were administered orally by inclusion in drinking water for 7 days. Pilot experiments were conducted to determine concentrations which did not diminish intake of water (monitored daily) or adversely affect health. Treatment of mice in the control, benzoate or cinnamate treatment groups (Figures 3 and 4) was started from 5 weeks of age in mice that were exposed pre‐natally to maternal administration of sodium formate (30 mg/mL) for the first 15 days of pregnancy in order to prevent structural malformations (neural tube defects or ventriculomegaly).^15^, ^36^

### 4.5 Glycine and guanidinoacetate quantification

Glycine and guanidinoacetate assays were performed using established methods in the Chemical Pathology Department of Great Ormond Street Hospital for Children NHS Foundation Trust. For tissues, concentration was normalised to protein content. Analysis of amino acids (glycine) was performed by cation‐exchange chromatography, with spectrophotometric detection at 570 nm following post‐column derivatisation with ninhydrin. Guanidinoacetate analysis was performed by liquid chromatography coupled to electrospray ionisation (ESI)‐tandem mass spectrometry, with a stable isotope labelled (2C^15^) internal standard (see Supporting Information for additional details).

### 4.6 Metabolite analysis (relative quantification)

Relative quantification of metabolites was performed by mass spectrometry by Metabolon (Morrisville, North Carolina). Samples were extracted at a constant mass to volume ratio with methanol precipitation of proteins. The sample extract was dried and reconstituted for analysis by ultra‐performance liquid chromatography coupled to tandem mass spectrometry (UPLC‐MS/MS). Each sample was analysed by each of four methods using reverse phase (RP) LC with negative ion mode ESI, hydrophilic interaction LC (HILIC; polar) with negative ion mode ESI, and two methods using reverse phase (RP) LC with positive ion mode ESI. A pooled matrix sample served as a technical replicate throughout the analysis. Compounds were identified by comparison to a library of authenticated standards and peaks were quantified using area‐under‐the‐curve (see Supporting Information for additional details).

Graphs show the data expressed as mass spectrometry counts, allowing relative quantitation within the data for each specific metabolite (as opposed to comparison between different metabolites which is not possible on the basis of raw counts owing to differential sensitivity and/or platforms used for each metabolite). Heatmapper^37^ was used for generation of heat‐maps and for cluster analysis (using Average Linkage and Euclidean distance measurement method).

### 4.7 Statistical analysis

Statistical analysis was performed using Sigmastat (v3.5, Systat Software) and Metabolon proprietary tools. Quantitative data was analysed by *t*‐test (two groups) or one‐way ANOVA (three or more groups) with Holm‐Sidak Pairwise comparison for post‐hoc analysis. Relative quantification of glycine and glycine derivatives was performed by pairwise comparison of groups using Welch's two sample *t*‐test. False discovery rate was estimated to take into account multiple comparisons. Raw data for specific metabolites was compared between groups by One‐Way ANOVA.

## CONFLICT OF INTEREST

Nicholas Greene consults for LifeMax Laboratories Inc and Andrew Copp acts as consultant for ViiV Healthcare Limited, with any fees going to support their research programmes. UCL, together with Nicholas D. E. Greene, have filed a patent covering aspects of the subject matter of this manuscript. The other authors declare that they do not have competing interests.

### AUTHOR CONTRIBUTIONS

Study design Kit‐Yi Leung, Nicholas D. E. Greene; Experimental work Kit‐Yi Leung, Sandra C. P. De Castro, Chloe Santos, Dawn Savery, Helen Prunty, Stuart Bennett, and Diana Gold‐Diaz; Data analysis and interpretation; Nicholas D. E. Greene, Kit‐Yi Leung, Simon Heales, and Andrew J. Copp; Writing and editing: Nicholas D. E. Greene, Kit‐Yi Leung, and Andrew J. Copp; All authors approved the final manuscript.

## Supporting information

**APPENDIX S1.** Supporting Information: Expanded Methods.

**APPENDIX S2.** Supporting Information: Figures.
