# Supplementary material for: Regulation of glycine metabolism by the glycine cleavage system and conjugation pathway in mouse models of non‐ketotic hyperglycinemia
Source: J Inherit Metab Dis. 2020 Aug 11;43(6):1186–98. doi: 10.1002/jimd.12295 (PMC8436753; doi:10.1002/jimd.12295)
Supplement: Supplementary file 1 — APPENDIX S1. Supporting Information: Expanded Methods. [file JIMD-43-1186-s001.pdf]

Materials and Methods

Mice

Animal studies were carried out under regulations of the Animals (Scientific Procedures) Act 1986 of the UK Government, and in accordance with the guidance issued by the Medical Research Council, UK in *Responsibility in the Use of Animals for Medical Research* (July 1993). Food was freely available throughout the study (standard breeder diet, Teklad).

Mice *Gldc*-deficient mice, denoted *Gldc*^{GT1}, carry a gene-trap construct in intron 2 of *Gldc* [1]. *Gldc* null (*Gldc*^{GT2}, denoted *Gldc*$^{-}$) carry a gene-trap construct in intron 19 [2]. Both strains were on a *C57BL/6J* genetic background. Mice were genotyped by PCR of genomic DNA.

Hepatocyte-specific targeting of the liver used transgenic mice expressing Cre recombinase under the control of the mouse albumin promoter and enhancer and three α-fetoprotein enhancers (*AlfpCre*) [3]. Conditional rescue of *Gldc* expression in *Gldc*^{GT1}-carrying mice was achieved by generation of *Gldc*$^{GT1/+}$; *AlfpCre/*+ mice followed by intercross with *Gldc*$^{GT1/+}$ to generate mice of genotype *Gldc*$^{GT1/GT1}$; *AlfpCre/*+.

The Gldc^{S956Y} mouse line (C57BL/6NTac-Gldc^{em1H}/H) was generated by the GEMM programme at MRC Harwell (A410) and is available through the European Mouse Mutant Archive (EMMA). Mice were generated by CRISPR/Cas9 targeting on a C57BL/6NTac genetic background by pronuclear injection in 1-cell stage embryos. Three nucleotide alterations were introduced into the *Gldc* locus. Genomic DNA was extracted from ear clip biopsies and the mutant allele was sequenced in both directions to confirm targeting. The S956Y mutation was encoded by a nucleotide substitution, c.3025C>A. Two synonymous variants were also introduced (c.3023C>T, c3029G>A) to allow genotyping and validation of targeting. The c.3029G>A variant generated an MseI site to enable genotyping by restriction digest of genomic PCR product. Both variants gave specificity to an allele-specific digital drop PCR (ddPCR) assay that was used to confirm single insertion of donor oligonucleotides in the genome. This assay showed 0 copies of the mutant allele in wild-type mice and 1 copy in the F1 heterozygotes. Another ddPCR assay that is universal for the Gldc gene (detects mutant and wild-type alleles) confirmed presence of 2 copies in mutant and wild-type mice. Heterozygous *Gldc*$^{S956Y/+}$ and *Gldc*$^{+/-}$ mice were intercrossed to generate *Gldc*$^{S956Y/-}$ offspring.

Sample collection

Blood was collected by cardiac puncture under isofluorane anaesthetic into lithium heparin tubes. Plasma was separated by centrifugation at 3,000 g at room temperature and stored at -20 °C. Tissues were collected immediately after sacrifice, rinsed in phosphate buffered saline (PBS), snap frozen on dry ice and stored at -80 °C (for RNA and metabolite analysis).

qRT-PCR

The abundance of *Gldc* mRNA in mice carrying the $Gldc^{GT1}$ allele was analysed by qRT-PCR. RNA was prepared using TRI reagent (Invitrogen), genomic DNA removed by DNase I digestion (DNA-free, Ambion) and first strand cDNA synthesis performed using random hexamers (Superscript VILO cDNA synthesis kit). The abundance of *Gldc* mRNA was analysed by qRT-PCR (iQ SYBR Green) on a CFX Real-Time PCR Detection System (Bio-Rad), with each sample analysed in triplicate. Primers were located in exons 2 and 4 (5'- AGCATTGATGAGCTCATCGAG and 5'-TCCAGCAGGGAAGCGTTGGC) of mouse *Gldc* to amplify the wild-type but not the mutant transcript. Results were normalised to abundance of *Gapdh* mRNA. Relative expression between individuals was determined by normalisation of each sample to one of the wild-type samples (set as 1.0).

Mouse treatments

Sodium benzoate and sodium cinnamate were administered orally by inclusion in drinking water for 7 days. In pilot experiments, inclusion of 35 mM benzoate, 35 mM cinnamate or 70 mM cinnamate did not significantly lower daily intake of drinking water compared with untreated water (n = 4 per group, monitored daily for 3 days). Water intake was significantly lower at benzoate concentration of 70 mM (25% reduction; $p<0.05$) or higher and treatment was halted.

Glycine quantification

Amino acid analysis was performed in the Chemical Pathology Department of Great Ormond Street Hospital for Children NHS Foundation Trust. Tissue was sonicated in phosphate buffered saline containing 1x protease inhibitor cocktail (Roche) and an aliquot removed for protein determination by Qubit assay. From plasma or tissue lysate, protein was precipitated

by addition of 5-sulphosalicylic acid containing a specified quantity of internal standard (S-(2-Aminoethyl)- L-cysteine hydrochloride (AEC)), in a ratio of 2:1 (sample: internal standard solution). Samples were precipitated for 30 minutes at 4°C, centrifuged and the supernatant removed and filtered prior to analysis. 60µL of the sample was injected onto a Lithium High Performance Physiological Column (cation-exchange) on a Biochrom 30 amino acid analyser. Amino acids were detected spectrophotometrically at 570 nm by post-column derivatisation with ninhydrin. For tissues, concentration was normalised to protein content.

Guanidinoacetate quantification

Analysis of guanidinoacetate was performed in the Chemical Pathology Department of Great Ormond Street Hospital for Children NHS Foundation Trust by liquid chromatography coupled to electrospray ionisation-tandem mass spectrometry (LC-ESI-MS/MS, based on [4]), using an Exion LC (AB Sciex) MDS SCIEX API4000 (Applied Biosystems). Samples comprised plasma or tissue lysate; tissue was sonicated in phosphate buffered saline containing 1x protease inhibitor cocktail (Roche). Guanidinoacetic acid ($2C^{15}$) internal standard was added to each sample and each assay included 5 internal quality control plasma samples. A 7-point calibration was performed for each run and the method is linear to at least 30 µM. Average imprecision (intra-batch CV) was 4.1% for urine and 6.6% for plasma. The assay is monitored by External Quality Assurance scheme performance.

Metabolite analysis by relative quantification

Mass spectrometry-based metabolite analysis was performed by Metabolon (Morrisville, NC, USA). Samples were extracted at a constant mass to volume ratio and prepared using a MicroLab STAR system (Hamilton Company) with proteins removed by methanol precipitation. The sample extract was dried and reconstituted in solvents compatible with one of four methods for analysis by ultra-performance liquid chromatography coupled to tandem mass spectrometry (UPLC-MS/MS) on an ACQUITY UPLC (Waters) and a Q-Exactive high resolution/accurate mass spectrometer interfaced with a heated electrospray ionization (HESI-II) source and Orbitrap mass analyzer (Thermo Scientific). Methods comprised (i) reverse phase LC using a C18 column with conditions optimised for negative ion mode electrospray ionisation (ESI); (ii) LC using a HILIC column (Waters UPLC BEH Amide 2.1x150 mm, 1.7 µm) with negative ion mode ESI; (iii) and (iv) using positive ion mode ESI following

elution from a C18 column (Waters UPLC BEH C18-2.1x100 mm, 1.7 µm) optimized for (iii) hydrophilic compounds using gradient elution from using water and methanol, containing 0.05% perfluoropentanoic acid (PFPA) and 0.1% formic acid (FA), or (iv) more hydrophobic compounds, using gradient elution with methanol, acetonitrile, water, 0.05% PFPA and 0.01% FA. In this study, guanidinoacetate, gamma-glutamylglycine, 3-methylcrotonylglycine, tigloylglycine, 3-hydroxybutyroylglycine, leucylglycine and glycylleucine were analysed in positive ion mode (condition iii), N-acetylglycine and propionylglycine in negative ion mode following HILIC (condition ii), and all other glycine metabolites in standard negative ion mode (condition i)

Quality control samples included a pool of human plasma which enabled evaluation of process specification and a pooled sample containing an aliquot from each individual sample which served as a technical replicate throughout the analysis. Raw data was extracted, peak-identified and QC processed using Metabolon's hardware and software. Compounds were identified by comparison to library entries of authenticated standards on the basis of the retention time/index (RI), mass to charge ratio (*m/z)*, and chromatographic data (including MS/MS spectral data). Peaks were quantified using area-under-the-curve. The median relative standard deviation was 3-4% for internal standards in brain and liver (measuring instrument variability), and 8% and 7-9% for the pooled samples from brain and liver, respectively (measuring total process variability).

Graphs were plotted showing mass spectrometry counts (raw data), which allows sample comparison within the data for each specific metabolite. Variation in scale between metabolites is partially dependent on the abundance but reflects other several factors including sensitivity/response so is indicative only. Heatmaps were generated using Heatmapper[5] using scaled data normalised across all the samples for a specific metabolite. Clustering of groups based on the heatmap profile was based on the Average Linkage clustering method using Euclidean distance as the distance measurement method.

Statistical analysis

Statistical analysis was performed using Sigmastat (v3.5, Systat Software) and Metabolon proprietary software. Quantitative data (mRNA, glycine and guanidinoacetate abundance)

was analysed by t-test (two groups) or ANOVA (three or more groups) with Holm-Sidak Pairwise comparison for post- hoc analysis. Prior to application of these methods data were checked for normal distribution.

For relative quantification (metabolome), we tested the hypothesis that glycine derivatives are altered in Gldc-deficient mice by initial pairwise comparison, of glycine/glycine derivatives present on the Metabolon panel, between liver of wild-type, Gldc-deficient and liver-rescued Gldc-deficient mice (Fig. 2) by Welch's two sample t-test ($p<0.05$ cut-off). Raw data (counts) for these metabolites were compared between groups by One-Way ANOVA, with false discovery rate of 5% (Benjamini-Hochberg procedure) for multiple testing correction. This list of differentially abundant metabolites was assessed for the effect of benzoate and cinnamate.

1. Pai, Y.J., *et al.* Glycine decarboxylase deficiency causes neural tube defects and features of non-ketotic hyperglycinemia in mice. *Nat. Commun* **6**, 6388 (2015).
2. Leung, K.Y., *et al.* Partitioning of One-Carbon Units in Folate and Methionine Metabolism Is Essential for Neural Tube Closure. *Cell Rep* **21**, 1795-1808 (2017).
3. Kellendonk, C., Opherk, C., Anlag, K., Schutz, G. & Tronche, F. Hepatocyte-specific expression of Cre recombinase. *Genesis* **26**, 151-153 (2000).
4. Carling, R.S., Hogg, S.L., Wood, T.C. & Calvin, J. Simultaneous determination of guanidinoacetate, creatine and creatinine in urine and plasma by un-derivatized liquid chromatography-tandem mass spectrometry. *Ann Clin Biochem* **45**, 575-584 (2008).
5. Babicki, S., *et al.* Heatmapper: web-enabled heat mapping for all. *Nucleic Acids Res* **44**, W147-W153 (2016).
