# Supplementary material for: Regulation of glycine metabolism by the glycine cleavage system and conjugation pathway in mouse models of non‐ketotic hyperglycinemia
Source: J Inherit Metab Dis. 2020 Aug 11;43(6):1186–98. doi: 10.1002/jimd.12295 (PMC8436753; doi:10.1002/jimd.12295)
Supplement: Supplementary file 2 — APPENDIX S2. Supporting Information: Figures. [file JIMD-43-1186-s002.pdf]

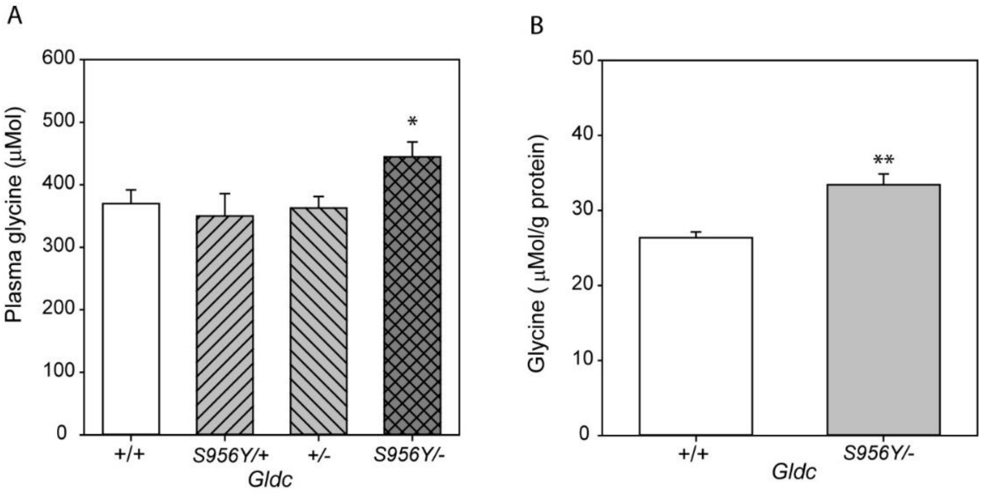

Supplementary Fig. 1 Elevated glycine concentration in plasma and brain of adult *Gldc*$^{S956Y/-}$ mice. (A) Plasma glycine in *Gldc*$^{+/+}$ (n = 6), *Gldc*$^{S956Y/+}$ (n = 2), *Gldc*$^{S956Y/+}$ (n = 5) and *Gldc*$^{S956Y/-}$ (n = 8). Note the significant elevation of glycine concentration in *Gldc*$^{S956Y/-}$ plasma. (B) Brain glycine shows moderate (1.3 fold) but significant accumulation in *Gldc*$^{S956Y/-}$ mice (n = 8) compared with wild-type (n = 4) littermates at 6 weeks of age (significant difference compared with wild-type **p<0.005 t-test; *P<0.05 ANOVA).

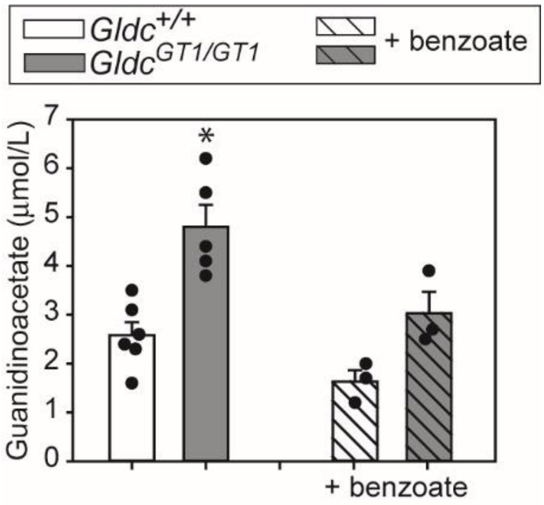

Supplementary Fig. 2. Plasma guanidinoacetate concentration after benzoate treatment

Plasma guanidinoacetate concentration is lowered by oral administration of benzoate for 7 days (* p<0.05, different from treated *Gldc*$^{GT1/GT1}$). Data for untreated mice are duplicated from Fig. 2P; these mice were maintained and analysed in parallel.

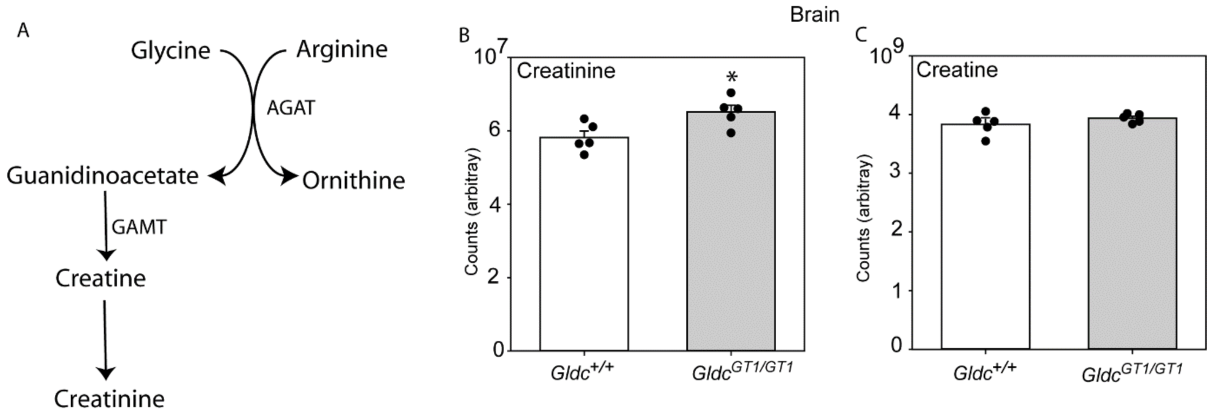

Supplementary Fig. 3. Creatinine and creatine abundance in brain of adult *Gldc*-deficient mice. (A) Guanidinoacetate is generated by the action of L-Arginine:glycine amidinotransferase (AGAT) and is a precursor in production of creatine (mediated by guanidinoacetate methyltransferase; GAMT) and creatinine. (B) Creatinine shows mild but significant increase in abundance in the brain of adult *Gldc*$^{GT1/GT1}$ mice compared with wildtypes, whereas (C) creatine did not differ between genotypes (significant difference compared with wild-type *p<0.05 t-test).
